# Major intracranial arterial stenosis influence association between baseline blood pressure and clinical outcomes after thrombolysis in ischemic stroke patients

**DOI:** 10.1002/brb3.3022

**Published:** 2023-05-22

**Authors:** Lijie Gao, Zuoxiao Li, Zhengzhou Yuan, Xingyang Yi, Jie Li, Chaohua Cui, Ning Chen, Li He

**Affiliations:** ^1^ Department of Neurology West China Hospital of Sichuan University Chengdu China; ^2^ Department of Neurology Affiliated Hospital of Southwest Medical University Luzhou China; ^3^ Department of Neurology People's Hospital of Deyang City Deyang China

**Keywords:** acute ischemic stroke, baseline blood pressure, intracranial artery stenosis, thrombolysis

## Abstract

**Background:**

This study aimed to investigate the relationship between baseline blood pressure (BP) and clinical outcomes after thrombolysis for acute ischemic stroke (AIS) in different intracranial arterial stenosis subgroups.

**Methods:**

AIS patients from multicenter with intravenous thrombolysis were retrospectively enrolled from January 2013 to December 2021. We categorized participants into severe (≥ 70%) and nonsevere (< 70%) stenosis of major intracranial arteries subgroups. The primary outcome was unfavorable functional outcome defined as 3‐month modified Rankin Scale (mRS) ≥2. The association coefficients between baseline BP and functional outcomes were estimated in general linear regression model. The interactive effect was tested to determine the influence of intracranial arterial stenosis on the association between BP and clinical outcomes.

**Results:**

A total of 329 patients were included. Severe subgroup was detected in 151 patients with average age of 70.5. Association between baseline diastolic BP (DBP) and unfavorable functional outcome in intracranial artery stenosis subgroups was significantly different (*p* for interaction < .05). In nonsevere subgroup, higher baseline DBP was associated with higher risk of unfavorable outcome (OR 1.11, 95% CI 1.03 to 1.20, *p* = .009) compared with severe subgroup (OR 1.02, 95% CI 0.97 to 1.08, *p* = .341). Besides, intracranial artery stenosis also modified association between baseline systolic BP (SBP) and 3‐month death (*p* for interaction < .05). In severe subgroup, higher baseline SBP was associated with decreased 3‐month death risk (OR 0.88, 95% CI 0.78 to 1, *p* = .044) compared with nonsevere subgroup (OR 1, 95% CI 0.93 to 1.07, *p* = .908).

**Conclusions:**

The major intracranial artery state modulates association between baseline BP and 3‐month clinical outcomes after intravenous thrombolysis.

## Graphical abstract

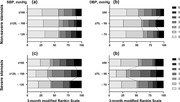

## 1 BACKGROUND

Thrombolysis is widely recommended as a standard therapy for acute ischemic stroke (AIS) (Adams et al., 2007; Herpich & Rincon, 2020; Powers et al., 2018). However, 40% to 60% of thrombolytic AIS patients do not attain favorable functional outcomes (Lees & Bluhmki, 2010). This circumstance indicates that there remains a major unmet medical need. It is well known that blood pressure (BP) during the acute stage of ischemic stroke is one of the most important influencing prognostic factors. Most guidelines for the management of ischemic stroke recommend controlling BP before thrombolysis to below 180–185/100–110 mmHg (Powers et al., 2018). However, the optimal level of BP is not well established. Generally, BP has been shown to have a U‐ or J‐shaped relationship with a beneficial clinical outcome. Both high and low BP indicate a poor prognosis (Keezer et al., 2008; Leonardi‐Bee et al., 2002). The major cerebral vascular state may modulate this association (Matusevicius et al., 2020). For example, in patients with nonsevere (< 70%) intracranial arterial stenosis, elevated SBP increased the risk of stroke recurrence. However, in those with severe (≥70%) stenosis, there was no such significant relevance (Turan et al., 2007). These results suggest that the vascular state, which can directly influence the hemodynamic response, may have an essential impact on the relationship between BP and the clinical outcome of AIS patients. However, the association between the BP level before thrombolysis and the curative effect of AIS patients with various vascular statuses remains unclear. This leaves an imprecise wide range of BP levels to the judgment of clinical neurologists. We aimed to investigate the potential association of BP levels before thrombolysis with functional outcomes in intravenous thrombolysis‐treated AIS patients with various degrees of intracranial arterial stenosis.

## 2 METHODS

### 2.1 Study design and population

This was a multicenter, retrospective, hospital‐based study. Consecutive patients with AIS who were treated with intravenous thrombolysis in the West China Hospital of Sichuan University, Affiliated Hospital of Southwest Medical University, and the People's Hospital of Deyang City between January 2013 and December 2021 were retrospectively enrolled. AIS was defined according to the World Health Organization criteria (The World Health Organization, 1999) and the Chinese guidelines (高峰, 徐安定, 2015). Thrombolysis was administered within 4.5 h (Alteplase) or 6 h (urokinase) after stroke onset according to widely used evidence‐based guidelines for the early management of AIS in China. We screened ICD codes to originally find acute ischemic stroke in each study center. All included patients were required to undergo emergency CT angiography (CTA) of the head and neck prior to initiating intravenous thrombolysis. Scanning systems were established in all three centers to perform brain imaging evaluations as soon as possible without delaying treatment.

Study inclusion criteria were as follows: (1) AIS patients with only thrombolysis but without endovascular therapy; (2) AIS patients fulfilled CTA examination of neck and head and had accessible intracranial artery data. Study exclusion criteria were as follows: (1) AIS patients with tandem or isolated extracranial arterial stenosis; (2) patients with unfavorable previous functional status (modified Rankin Scale (mRS) (Adams et al., 1999) score ≥ 2 before the present stroke); (3) AIS patients with missing BP data; (4) AIS patients lost 3‐month follow‐up.

The study was approved by the Institutional Ethics Committee of West China Hospital, Affiliated Hospital of Southwest Medical University, and the People's Hospital of Deyang City, Sichuan University (approval number 2019 [319]). The data were anonymized, and the requirement for informed consent was waived.

### 2.2 BP measurements

Certified trained nurses were authorized to obtain the BP for each patient. They used arm mercury or adjusted digital sphygmomanometers to obtain the BP of study patients. In the three institutions of our study. According to the protocol of the stroke units in the three institutions of our study, BP was first measured in the emergency department once a potential patient arrived. It was evaluated and recorded every 15 min prior to thrombolysis. If the BP was found to be over 180/100 mmHg in a patient eligible for thrombolysis, antihypertensive treatments, such as Labetalol and Nicardipine, were cautiously administered according to Chinese stroke guidelines. In the present study, baseline BP was defined as the last BP measured before the initiation of thrombolysis treatment.

### 2.3 Data collection

Demographic information and clinical data were collected during the admission of each patient from medical records. This included age, sex, body mass index (BMI), smoking status, disease history (i.e., previous stroke, hypertension, diabetes, atrial affiliation and coronary heart disease), stroke subtypes, door‐to‐needle time (DNT), laboratory tests, interventions, complications, and imaging findings. The data collectors were not aware of the study design. Stroke severity was assessed using the National Health Institute Stroke Scale Score (NIHSS) (Adams et al., 1999). Hemorrhagic transformation was defined as any hemorrhage finding by brain CT within 7‐day after thrombolysis with or without any symptomatic deterioration.

Stenosis of the major intracranial arteries relevant to new onset ischemic stroke was assessed using CTA performed before thrombolysis. The major intracranial arteries mainly comprise the anterior cerebral artery (ACA), M1 and M2 segments of the middle cerebral artery (MCA), posterior cerebral artery (PCA), basilar artery, intracranial segment of the internal carotid artery (ICA) and vertebral artery. Two neurologists assessed the vascular status independently, and discrepancies were resolved by discussion. Degrees of stenosis were recorded using the formula (normal proximal diameter‐stenosis diameter)/normal proximal diameter × 100% (Bartlett et al., 2006). If there were tandem lesions or multiple stenoses in the large intracranial arteries, the most severe stenosis site was considered in the analysis. In the present study, according to previous classification criteria (Kasner et al., 2006; Turan et al., 2007) and clinical routines, all participants were grouped according to the degree of artery stenosis. They were categorized into the severe stenosis group (stenosis degree ≥70%) or the nonsevere stenosis group (stenosis degree < 70%).

TOAST classification was determined according to onset symptoms, disease history, bedside electronic monitoring, and head images by receiving resident physicians. Patients with cardiac embolism or other causes of stroke but not severe major artery stenosis were assigned to the nonsevere stenosis group. Once severe stenosis was detected in such patients, patients with irrelevant stenosis were still assigned to nonsevere stenosis group; and to severe stenosis group with relevant stenosis.

### 2.4 Follow‐up and outcomes

Patients were followed by face‐to‐face assessments in the outpatient department of each hospital or by telephone interviews. The primary outcome was the 3‐month mRS score ≥2. The secondary outcomes were (1) all‐cause mortality within 3 months, (2) NIHSS score on the 1st and (3) discharge after thrombolysis treatment. Outcomes were evaluated by trained neurologists who were blinded to the patients’ baseline BP and cerebral vascular status. The baseline information and follow‐up information were obtained by independent neurologists, in which neurologists responsible for follow‐up were unaware of the corresponding patients’ baseline BP or cerebral vascular stenosis or the study design and purpose.

### 2.5 Statistical analyses

Data were presented as the mean ± standard deviation (SD) for continuous variables and as frequencies and percentages for categorical variables. Student's *t*‐tests for continuous variables and chi‐square tests for categorical variables were applied to assess differences in clinical characteristics. If the frequency of categorical variables was less than 10, Fisher's exact probability test was used to calculate differences. To assess 3‐month mRS distribution in different intracranial artery stenosis by baseline BP subgroups, we classified the BP into tertile group. To evaluate the independent effect of BP on outcomes, we further adjusted the analyses for age, sex, smoking, BMI, TOAST, DNT, NIHSS score before thrombolysis, disease history (diabetes, hypertension, coronary heart disease, atrial fibrillation and stroke), total cholesterol, and triglyceride. These confounding variables were included based on previous reports (Berge et al., 2015; Goyal et al., 2017; Rusanen et al., 2015) and clinical experience. The correlation coefficients (β/OR) and 95% CIs between baseline BP and functional outcomes of AIS patients treated with intravenous thrombolysis across varying degrees (< 70% or ≥ 70%) of major intracranial arterial stenosis were evaluated with linear/logistic regression. These interactions were evaluated both in general linear and quadratics models. A *p* value < .05 was considered to be statistically significant in all analyses. The data were analyzed using R software (version 4.0.1; Vienna, Austria; https://www.R‐project.org/).

## 3 RESULTS

### 3.1 Characteristics of study patients in different major intracranial artery status

**FIGURE 1:**
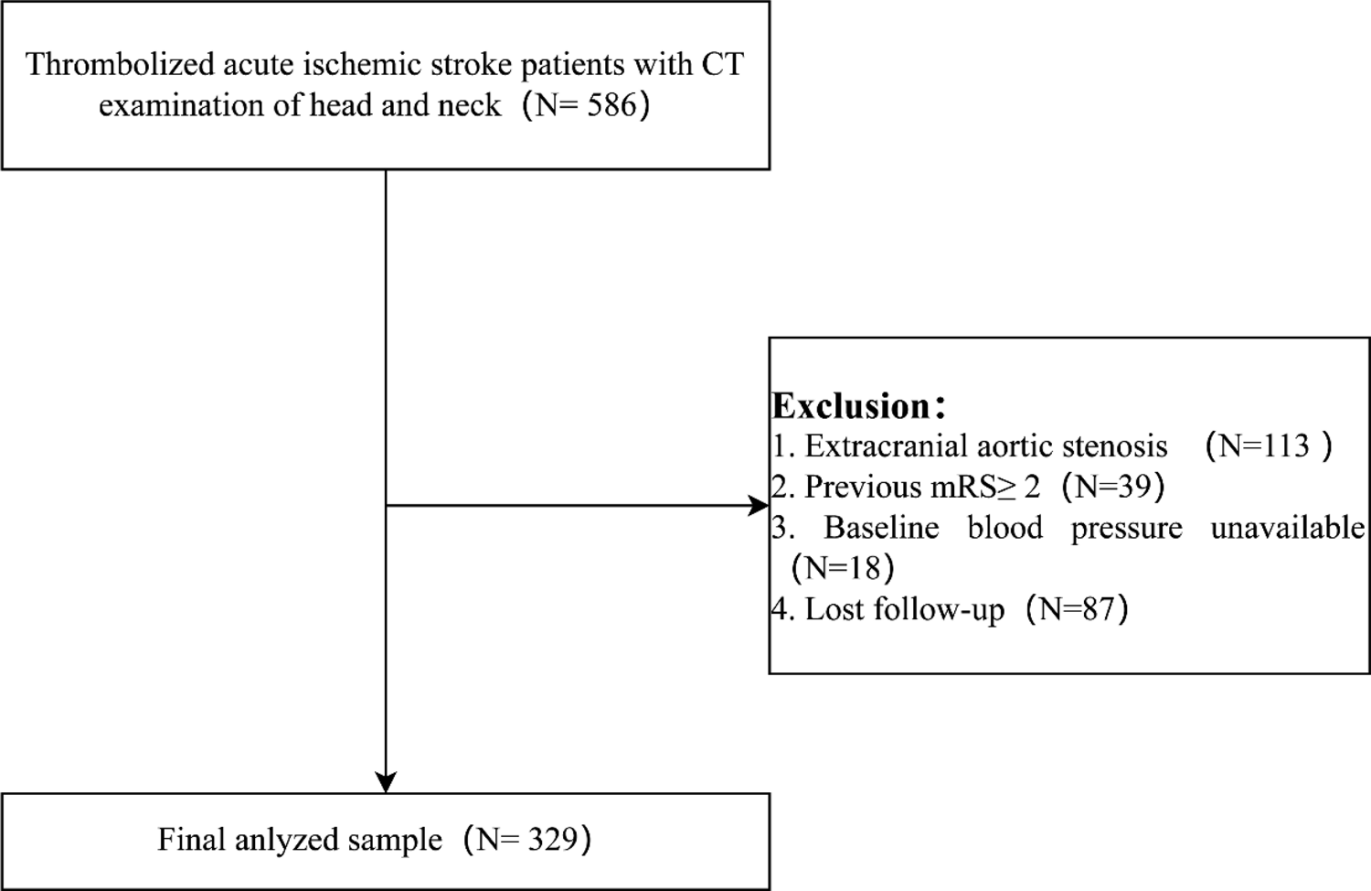
Study flowchart. CT, computed tomography, mRS, modified Rankin Scale.

A total of 586 AIS patients treated with intravenous thrombolysis had performed CTA of neck and head in all three centers during study period. We excluded 113 patients with extracranial arterial stenosis. Thirty‐nine patients had an unfavorable functional status before the current stroke, and 18 were with unavailable baseline BP. We further excluded 87 patients lost follow‐up, leaving 329 patients for final analyses (Figure 1).

The baseline clinical characteristics of study patients and each group with various intracranial artery stenosis are presented in Table 1. Mean age of the included patients was 70 ± 7.8 years, and 48.3% were men. Among the participants, severe stenosis (≥70%) of the major intracranial artery was detected in 151 patients, and 178 in nonsevere stenosis subgroup. The internal carotid and middle cerebral arteries were significantly higher in the severe stenosis subgroup. The distribution of etiological type was significantly different between the groups according to TOAST classification (*p* < .001). Stroke caused by large artery atherosclerosis was the most common in both groups. However, it was noted other etiology, such as cardioembolism, were also observed in severe subgroup. This might be due to the time point of major intracranial artery status assessment was before thrombolysis. Patients in the severe stenosis group had more serious neurological impairment on admission than those in the nonsevere stenosis group (mean NIHSS severe vs. nonsevere 9.9 vs. 8.2, *p* = .014).

**TABLE 1:** Baseline characteristics of study participants.

	Total	Intracranial artery stenosis		
	(*N* = 329)	Nonsevere	Severe	*p*
		<70%	≥70%	
		(*N* = 178)	(*N* = 151)	
Male, *n* (%)	159 (48.3)	88 (49.4)	71 (47)	.9
Age, years, mean ± SD	70.0 ± 7.8	69.0 ± 8.6	70.5 ± 7.2	.372
BMI, kg/m2, mean ± SD	23.2 ± 5.6	23.3 ± 5.7	23.1 ± 5.4	.853
Smoke, *n* (%)	103 (31.3)	48 (27)	55 (36.4)	.056
Disease history, *n* (%)				
Stroke	18 (8.7)	8 (7)	10 (10.6)	.499
Hypertension	188 (57.1)	100 (56.2)	88 (58.3)	.786
Diabetes	67 (20.4)	34 (19.1)	33 (21.9)	.631
Atrial fibrillation	93 (28.3)	44 (24.7)	49 (32.5)	.192
Coronary artery disease	33 (10.0)	18 (10.1)	15 (9.9)	.999
TOAST, *n* (%)				<.001*
LAA	144 (43.8)	52 (29.2)	92 (60.9)	
CE	88 (26.7)	46 (25.8)	42 (27.8)	
SAA	53 (16.1)	49 (27.5)	4 (2.6)	
SOE	4 (1.2)	4 (2.2)	0 (0)	
SUE	40 (12.2)	27 (15.2)	13 (8.6)	
Occlusion artery, *n* (%)				
ICA	114 (34.7)	46 (25.8)	68 (45)	<0.001^*^
MCA	47 (37.3)	12 (17.9)	35 (59.3)	<0.001^*^
ACA	10 (3.0)	4 (2.2)	6 (4)	0.774
PCA	9 (2.7)	3 (1.7)	6 (4)	0.38
Vertebrobasilar artery	27 (8.2)	9 (5.1)	18 (11.9)	0.112
NIHSS before thrombolysis, mean ± SD	9.0 ± 6.3	8.2 ± 6.2	9.9 ± 6.2	0.014^*^
DNT, mean ± SD	66.0 ± 24.9	64.9 ± 24.9	67.4 ± 24.9	0.515
IV urokinase, *n* (%)	8 (0.0)	5 (0.0)	3 (0.0)	0.614
Total cholesterol, mmol/L, mean ± SD	4.5 ± 1.1	4.5 ± 1.1	4.5 ± 1.0	0.595
Triglycerides, mmol/L, mean ± SD	1.7 ± 1.3	1.9 ± 1.4	1.6 ± 1.0	0.065
Baseline SBP, mean ± SD	148.2 ± 26.4	147.4 ± 26.2	149.1 ± 26.8	0.562
Baseline DBP, mean ± SD	86.1 ± 16.6	86.4 ± 16.9	85.8 ± 16.3	0.752

* *p* < .05 (cervicocerebral stenosis < 70% vs. ≥70%).

Abbreviations: BMI, body mass index; CE, cardioembolism; DNT, door‐to‐needle time; IV, intravenous; LAA, large artery atherosclerosis; NIHSS, National Institutes of Health Stroke Scale; SAA, small‐artery occlusion; SD, stand deviation; SOE, stroke of other determined cause; SUE, stroke of undetermined cause; TOAST, Trial of ORG 10172 in Acute Stroke Treatment.

The clinical outcomes of thrombolysis were shown in Table 2. The short‐term outcomes of thrombolysis were worse in severe stenosis of major intracranial artery patients. The mean NIHSS score at 24‐h after was 8.3 in severe subgroup and 6.4 in nonsevere subgroup (*p* = .019). For NIHSS score at discharge, the mean in severe subgroup was 8.8 and nonsevere subgroup was 5.8 (*p* = .002). But the distribution of hemorrhage transformation and long‐term outcomes measured by 3‐month mRS in major intracranial artery status subgroups were not significantly different.

**TABLE 2:** Thrombolysis outcomes of study participants.

	Total	Intracranial artery stenosis		
	(*N* = 329)	Nonsevere	Severe	*p*
		<70%	≥70%	
		(*N* = 178)	(*N* = 151)	
NIHSS 24‐h after thrombolysis, mean ± SD	7.3 ± 7.2	6.4 ± 7.0	8.3 ± 7.4	.019*
NIHSS at discharge, mean ± SD	7.2 ± 9.1	5.8 ± 8.0	8.8 ± 10.0	.002^*^
Hemorrhage transformation, *n* (%)	36 (10.4)	15 (8)	21 (13.1)	.168
3‐month mRS, *n* (%)				.299
0	82 (24.9)	52 (29.2)	30 (19.9)	
1	92 (28.0)	51 (28.7)	41 (27.2)	
2	23 (7.0)	14 (7.9)	9 (6)	
3	49 (14.9)	21 (11.8)	28 (18.5)	
4	29 (8.8)	15 (8.4)	14 (9.3)	
5	17 (5.2)	8 (4.5)	9 (6)	
6	37 (11.2)	17 (9.6)	20 (13.2)	
3‐month death, *n* (%)	37 (11.2)	17 (9.6)	20 (13.2)	.378
3‐month mRS≥2, *n* (%)	155 (47.1)	75 (42.1)	80 (53)	.064

* *p* < .05 (cervicocerebral stenosis < 70% vs. ≥70%).

Abbreviations: mRS, modified Rankin Scale; NIHSS, National Institutes of Health Stroke Scale; SD, stand deviation.

### 3.2 Impact of major intracranial arterial stenosis on associations of baseline BP and outcomes

**FIGURE 2:**
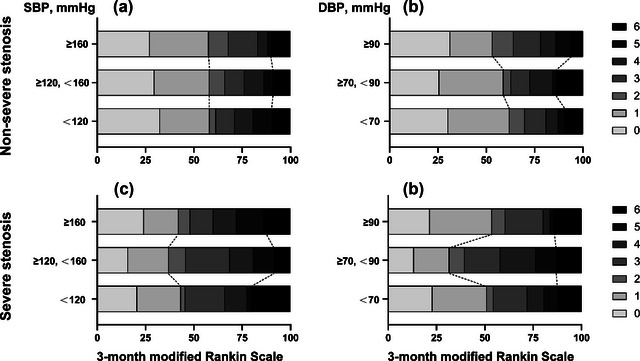
Three‐month mRS distribution in different intracranial artery stenosis by blood pressure subgroups. Baseline blood pressure was categorized into three groups according to tertile point. The SBP classified by 120 and 160 mmHg; while DBP by 70 and 90 mmHg. The panels (A) and (B) showed the 3‐month mRS distribution in different intracranial artery stenosis by SBP subgroups; panels (C) and (D) were by DBP subgroups. SBP, systolic blood pressure; DBP, diastolic blood pressure.

Baseline BP was classified into tertile groups. The distribution of 3‐month mRS scale in major intracranial artery status by baseline BP subgroups was shown in Figure 2. Tables 3 and 4 further showed different association between baseline SBP, DBP, and clinical outcomes among major intracranial artery stenosis subgroups. The association between baseline SBP and NIHSS score at 24‐h after thrombolysis, NIHSS at discharge did not differ in major intracranial artery stenosis subgroups (*p* for interaction > .05). Although, major intracranial artery stenosis influenced association between baseline SBP and unfavorable functional outcome (3‐month mRS score≥ 2) whether in general linear model or quadratics model (*p* for interaction < .05). Baseline SBP did not significantly associated with unfavorable functional outcome in nonsevere (OR 1.03, 95% CI 0.99 to 1.07, *p* = .113) or severe (OR 0.99, 95% CI 0.96 to 1.03, *p* = .643) major intracranial artery stenosis group. The association between baseline SBP and 3‐month death was significantly different in major intracranial artery stenosis subgroups (*p* for interaction < .05). In nonsevere subgroup, baseline SBP was not significantly associated with 3‐month death (OR 1, 95% CI 0.93 to 1.07, *p* = .908); in severe subgroup, baseline SBP negatively associated with 3‐month death (OR 0.88, 95% CI 0.78 to 1, *p* = .044).

**TABLE 3:** Association between baseline SBP and thrombolysis outcomes in different intracranial artery stenosis.

	Unadjusted model		Adjusted model		interaction *P*1^§^	interaction *P*2^†^
	OR/β (95% CI)	*p*	OR/β (95% CI)	*p*		
**Total population**	1 (0.99 to 1.01)	.861	1.01 (0.99 to 1.03)	.408		
**NIHSS 24‐h after thrombolysis**					.679	.079
Nonsevere intracranial artery stenosis	−0.01 (−0.05 to 0.04)	.791	−0.02 (−0.08 to 0.05)	.63		
Severe intracranial artery stenosis	−0.03 (−0.09 to 0.02)	.242	0.03 (−0.08 to 0.13)	.635		
**NIHSS at discharge**					.678	.312
Nonsevere intracranial artery stenosis	−0.02 (−0.07 to 0.03)	.433	−0.05 (−0.17 to 0.08)	.472		
Severe intracranial artery stenosis	−0.05 (−0.13 to 0.03)	.204	0.07 (−0.23 to 0.36)	.663		
**3‐month mRS≥2**					.004^*^	.001^*^
Nonsevere intracranial artery stenosis	1 (0.99 to 1.02)	.559	1.03 (0.99 to 1.07)	.113		
Severe intracranial artery stenosis	0.99 (0.98 to 1.01)	.33	0.99 (0.96 to 1.03)	.643		
**3‐month death**					.022^*^	.023^*^
Nonsevere intracranial artery stenosis	1 (0.98 to 1.02)	.824	1 (0.93 to 1.07)	.908		
Severe intracranial artery stenosis	0.99 (0.97 to 1.01)	.269	0.88 (0.78 to 1)	.044^*^		

^§^ *p* for interaction was calculated from linear or logistic regression model.

^†^ *p* for interaction was calculated from quadratics regression model.

^*^ *p* < .05.

Age, sex, smoking, BMI, TOAST, DNT, NIHSS score before thrombolysis, disease history (diabetes, hypertension, coronary heart disease, atrial fibrillation and stroke), total cholesterol, and triglycerides were adjusted in adjusted model.

Abbreviations: CI, confidence interval; mRS, modified Rankin Scale; NIHSS, National Institutes of Health Stroke Scale; OR, odds ratio.

**TABLE 4:** Association between baseline DBP and thrombolysis outcomes in different intracranial artery stenosis.

	Unadjusted model		Adjusted model		interaction *p*1^§^	interaction *p*2^†^
	OR (95% CI)	*p*	OR (95% CI)	*p*		
**Total population**	1 (0.99 to 1.02)	.597	1.04 (1 to 1.07)	.029^*^		
**NIHSS 24‐h after thrombolysis**					.922	.046^*^
Nonsevere intracranial artery stenosis	−0.02 (−0.1 to 0.07)	.704	−0.03 (−0.19 to 0.13)	.725		
Severe intracranial artery stenosis	−0.01 (−0.1 to 0.09)	.89	0.04 (−0.08 to 0.16)	.49		
**NIHSS at discharge**					.786	.316
Nonsevere intracranial artery stenosis	0 (−0.09 to 0.09)	.978	−0.07 (−0.38 to 0.23)	.647		
Severe intracranial artery stenosis	−0.01 (−0.13 to 0.12)	.93	0.04 (−0.33 to 0.42)	.824		
**3‐month mRS≥2**					.005^*^	.015^*^
Nonsevere intracranial artery stenosis	1.01 (0.99 to 1.04)	.327	1.11 (1.03 to 1.2)	.009^*^		
Severe intracranial artery stenosis	1 (0.97 to 1.02)	.808	1.02 (0.97 to 1.08)	.341		
**3‐month death**						
Nonsevere intracranial artery stenosis	1 (0.96 to 1.04)	.867	1.04 (0.92 to 1.17)	.507	.167	.086
Severe intracranial artery stenosis	0.99 (0.95 to 1.02)	.497	0.95 (0.85 to 1.06)	.335		

^§^ *p* for interaction was calculated from linear or logistic regression model.

^†^ *p* for interaction was calculated from quadratics regression model.

^*^ *p* < .05.

Age, sex, smoking, BMI, TOAST, DNT, NIHSS score before thrombolysis, disease history (diabetes, hypertension, coronary heart disease, atrial fibrillation, and stroke), total cholesterol, and triglycerides were adjusted in adjusted model.

Abbreviations: CI, confidence interval; mRS, modified Rankin Scale; NIHSS, National Institutes of Health Stroke Scale; OR, odds ratio.

For baseline DBP, major intracranial artery stenosis did not modify association between baseline DBP and NIHSS score or 3‐month death. But in nonsevere intracranial artery stenosis, baseline DBP was positively associated with unfavorable outcome (OR 1.11, 95% CI 1.03 to 1.2, *p* = .009); in severe subgroup this association was not significant (OR 1.02, 95% CI 0.97 to 1.08, *p* = .341). And major intracranial artery stenosis modified association between baseline DBP and unfavorable outcome (*p* for interaction < .05).

## 4 DISCUSSION

In this study, we explored the influence of major intracranial stenosis on association between baseline BP and clinical outcomes in AIS patients who received intravenous thrombolysis. Our findings suggest that the pretreatment vascular state of major intracranial arteries can influence the association between baseline BP and clinical outcomes 3 months after a stroke. Compared to nonsevere arterial stenosis, higher baseline SBP tended to be associated with lower risk of worse clinical outcomes in severe subgroup. And higher baseline DBP tended to be associated with higher risk of worse clinical outcomes in nonsevere subgroup than severe subgroup.

The interaction effects between intracranial arterial stenosis degrees and baseline BP on 3‐month clinical outcomes further support the hypothesis that arterial status is an essential modulating factor. Arterial status could be assessed carefully prior to thrombolysis, and the BP might be adjusted accordingly. Elevated BP is very common in the acute phase of ischemic stroke in two‐thirds to three‐quarters of patients (Saver, 2014). One of the reasons is that hypertension is the most important risk factor for stroke (Lees & Bluhmki, 2010) and is often undiagnosed or untreated prior to a cerebrovascular event (Saver, 2014). In our study, more than half of the included patients had a history of hypertension, with or without other risk factors, similar to most published ischemic stroke cohorts. Other reasons for early hypertension after stroke may include a comorbidity of stressful events and a compensatory change that maintains cerebral blood flow in the ischemic brain (Gąsecki et al., 2018). The management of BP elevation in the early acute phase remains a major unresolved issue.

Pathophysiologically, excessive BP accompanied by hyperperfusion may result in hemorrhage transformation and brain edema, while low BP along with hyporeperfusion could harm the recovery of the ischemic penumbra (Heiss et al., 2004; Vitt et al., 2019). Previous studies has reported a U‐shaped or J‐shaped relationship between BP values in the acute phase after stroke and functional outcome was always found (Keezer et al., 2008; Leonardi‐Bee et al., 2002; Martins et al., 2016). Therefore, we performed interaction effect in both linear and quadratics model. However, the optimal BP level that should be maintained to ensure the best outcome remains unknown and future studies concerned on this topic are needed. Besides, previous studies have reported different effect of SBP and DBP on cardiovascular outcomes (Flint et al., 2019). In general, higher SBP and higher DBP were associated with worse cardiovascular outcome. In our study, higher SBP or DBP was associated with worse outcome in nonsevere group. But the statistical significance was different in groups (SBP/outcomes vs. DBP/outcomes). Future larger prospective cohort or even RCT studies are needed to confirm our finding

Cerebrovascular patency status may be one of the most important factors that must be considered. This directly affects cerebral perfusion and autoregulation. Martins and colleagues found that in patients with AIS treated with intravenous thrombolysis or intraarterial therapies, there was a linear correlation between systolic BP and functional outcome in successfully recanalized patients. There was a J shape in patients with unsuccessful recanalization (Martins et al., 2016). Our study found that intracranial arterial stenosis before thrombolysis could also influence association between baseline BP and clinical outcomes.

Intracranial stenosis is one of the most common causes of ischemic stroke worldwide, with the highest occurrence in Asian, African, and Hispanic populations (Krasteva et al., 2020). Artery‐to‐artery embolism and obstruction of small penetrating arteries are the most important mechanisms of stroke (Krasteva et al., 2020). Hypoperfusion and hemodynamic abnormalities may be present in high‐grade intracranial arterial stenosis (Caplan et al., 2006; Krasteva et al., 2020). In AIS patients with nonsevere intracranial arterial stenosis (including other stroke causes in addition to nonsevere LAA), cerebral vascular hemodynamics in the relevant territory may be mildly affected. Under these conditions, excessive BP is more likely to elevate cerebral blood flow and cause hyperperfusion after thrombolytic treatment. Moreover, the impact of elevated BP on functional outcomes in such patients may be due mainly to the effects of BP on the progression of arterial lesions. This is a long‐term effect. These patients can benefit more from controlling BP to a relatively lower level prior to initiating intravenous thrombolysis. In contrast, in patients with severe intracranial arterial stenosis, perfusion in the relevant territory is seriously inadequate. These patients may be more susceptible to hypoperfusion and require relatively higher BP to improve the recovery of blood supply to the ischemic penumbra (Martins et al., 2016; Turan et al., 2007). As a result, either too high or too low BP can lead to poor outcomes due to various mechanisms. Therefore, a moderate level of BP may be most appropriate for such patients. However, the optimal BP range was not shown in the present study due to an insufficient study sample.

The association between baseline BP and short‐term neurological function was not modified by intracranial arterial stenosis. This may be because a total of 22 patients who died during the first week after stroke were not included in the 1‐day and 7‐day NIHSS assessments. This could impact the results and must be analyzed in further studies. Given the small number of patients with extremely high BP (all were treated if BP was higher than 180/100 mmHg before thrombolysis), the inability to demonstrate a difference between the incidences of hemorrhagic transformation between groups might be due to a lack of statistical power.

The percentage of any intracranial hemorrhage (10.4%) after thrombolysis is similar to that in previous studies (Seet & Rabinstein, 2012; Van Leyen et al., 2019; Wardlaw et al., 2014). The number was small in each group; therefore, the relationships between the occurrence of symptomatic intracranial hemorrhage and baseline BP or vascular status were not analyzed in the present study.

Zhou et al. (2021) conducted a secondary analysis of ENCHANTED cohort, reporting that intracranial stenosis did not modified the association between blood pressure lowering and outcomes. However, we found that intracranial stenosis did affect association between baseline SBP and 3‐month death and the association between baseline DBP and 3‐month unfavorable outcome. Possible reasons responsible for the difference included: (1) The ENCHANTED study was a secondary analysis of data from randomized controlled trials, while this study retrospectively collected data from patients with intravenous thrombolytic therapy from real world. (2) The blood pressure of the intervention group in the above study was controlled to be less than 140 mmHg, and the mean baseline systolic blood pressure of the thrombolytic patients in this study was higher. (3) The criteria for grouping the degree of intracranial arterial stenosis in the above studies were three groups: light, medium, and severe, which may require a larger sample size to verify the hypothesis. However, due to the sample size, this study only categorized stenosis into nonsevere and severe subgroups.

Our study was based on a hospital database, which may introduce the potential for bias and have certain limitations. First, this was a retrospective multicenter study, which lacked a standardized treatment protocol. CTA before thrombolysis was not mandatory according to AIS guidelines. Thus, the CTA implementation varied among hospitals. We excluded a relatively large number of patients without neck or head CTA. This would produce selection bias. Thus, further well‐designed prospective studies are needed to verify our findings. In addition, according to the guidance of AIS management, baseline BP was controlled to less than 180/100 mmHg. This resulted in the omission of conditions when BP was extremely high. Furthermore, we analyzed certain values of BP before thrombolysis but not variations in BP, leaving the accumulation effect of BP to be analyzed. Besides, we did not include patients with endovascular therapy. Our finding could not apply to AIS patients with endovascular therapy. Further studies were needed to estimate the modification effect of artery status on association between BP and clinical outcomes. Moreover, the symptomatic or asymptomatic arterial stenosis and recanalization status after treatment, which can have various influences on the perfusion status after thrombolysis, were not identified or grouped in our study. Further studies should investigate tandem vascular lesions (including the vascular status of extracranial arteries), and the impact of recanalization status should simultaneously be investigated.

Despite these limitations, this study supported the hypothesis that the impact of baseline BP on the functional outcome of AIS patients treated with intravenous thrombolysis was dependent on the degree of major intracranial arterial stenosis. Cerebral arterial status should be carefully considered when developing an individualized BP management plan before initiating thrombolysis in patients with AIS. In future studies, investigators must be aware of the need to assess arterial stenosis when exploring the optimal target BP level in potential patients undergoing thrombolytic treatment.

## 5 CONCLUSION

The major intracranial artery state modulates the association between baseline BP and 3‐month clinical outcomes in patients with AIS after intravenous thrombolytic treatment. Further studies are required to confirm these findings.

## AUTHOR CONTRIBUTIONS

H.L. and C.N. contributed conception and design of study. G.L.J. collected, analyzed, and interpreted the data. G.L.J. wrote the draft manuscript. All authors reviewed and helped revise the manuscript.

## CONFLICT OF INTEREST STATEMENT

The authors declare that they have no conflicts of interest.

### ETHICS STATEMENT

This study was approved by the Institutional Ethics Committee of West China Hospital of Sichuan University, Affiliated Hospital of Southwest Medical University, and the People's Hospital of Deyang City (approval number 2019 [319]). The data were anonymized, and the requirement for informed consent was waived by the Institutional Ethics Committee of West China Hospital of Sichuan University, Affiliated Hospital of Southwest Medical University, and the People's Hospital of Deyang City.

### PEER REVIEW

The peer review history for this article is available at https://publons.com/publon/10.1002/brb3.3022.

## Data Availability

Data were available with the appropriate study purpose to the corresponding author.
